# Fear of Recurrence in Advanced Cancer Patients: Sociodemographic, Clinical, and Psychological Correlates

**DOI:** 10.3390/cancers16050909

**Published:** 2024-02-23

**Authors:** Caterina Calderon, Marina Gustems, Rocio Galán-Moral, Maria M. Muñoz-Sánchez, Lorena Ostios-García, Paula Jiménez-Fonseca

**Affiliations:** 1 Faculty of Psychology, University of Barcelona, 08007 Barcelona, Spain; 2 Department of Medical Oncology, Hospital General Universitario de Ciudad Real, 13005 Madrid, Spain; 3 Department of Medical Oncology, Hospital General Virgen de la Luz, 16002 Cuenca, Spain; 4 Department of Medical Oncology, Hospital La Paz, 28046 Madrid, Spain; 5 Department of Medical Oncology, Hospital Universitario Central de Asturias, Instituto de Investigación del Principado de Asturias (ISPA), 33011 Oviedo, Spain

**Keywords:** advanced cancer, fear of recurrence, uncertainty, social support, depression, psychological factors

## Abstract

**Simple Summary:**

This study explores the fear of cancer recurrence in patients with advanced cancer undergoing systemic treatment, examining its association with sociodemographic and psychological factors. A multicenter study was conducted across 15 oncology departments in Spain, involving 1195 participants. Two groups based on fear levels were identified: 28% with low levels and 72% with high levels. High fear was associated with being female, being younger, having lower education, and having worse survival estimates, as well as increased depression, anxiety, somatic symptoms, uncertainty, and stronger social support. The findings highlight the need for targeted interventions and specific support for advanced cancer patients.

**Abstract:**

Fear of cancer recurrence significantly impacts advanced cancer patients, prompting emotional distress and increased healthcare utilization. This present study aims to analyze the fear of recurrence among patients with advanced cancer undergoing systemic treatment and its relationship with sociodemographic, clinical, and psychological factors. A multicenter cross-sectional study was conducted in 15 oncology departments across Spain, involving patients with locally advanced, unresectable, or metastatic cancer eligible for systemic treatment. Participants provided demographic information and completed instruments such as the Cancer Worry Scale, Brief Symptom Inventory, Mishel Uncertainty in Illness Scale, and the Duke–UNC-11 Functional Social Support Questionnaire (DUFSSQ). A total of 1195 participants participated: median age 66, 56% male, mostly metastatic cancers (80%), and common tumor sites. Two fear groups emerged: 28% low and 72% high levels of fear. High fear was associated with being female, being younger, lower levels of education, and worse survival estimates. High fear correlated with more depression, anxiety, somatic symptoms, uncertainty, and stronger social support. Multivariate analyses indicated that younger patients, those with shorter survival estimates, higher depression and anxiety scores, more uncertainty, and stronger social support had a greater likelihood of experiencing fear of recurrence, while the opposite was true for older patients. This study underscores distinct fear of recurrence profiles in advanced cancer patients, emphasizing the need for targeted interventions and support. Future research should delve deeper into understanding their repercussions for improving patient care and well-being.

## 1. Introduction

Fear of cancer progression, also known as fear of recurrence [1,2], is a primary concern among cancer patients, particularly those with advanced cancer [3]. Evidence suggests that 20–70% of patients experience significant levels of this phenomenon [4]. While a certain degree of fear of recurrence can be adaptive, very high levels are associated with anxiety, depression, and impaired quality of life, as well as increased use of anxiolytic and antidepressant medications [5,6] and healthcare services’ utilization [4,7].

Several potential risk factors related to fear of cancer recurrence have been studied in patients [4]. These factors include demographic characteristics such as age, sex, marital status, educational level, and income [8,9]. Younger age has consistently been associated with greater fear of recurrence; however, findings regarding other demographic factors have been mixed [3,4,10]. Some studies have reported that women experience higher levels of fear of recurrence than men [9,11], while others have found greater concern among men, especially for specific types of cancer [12,13], or no significant association between gender and fear of recurrence at all [14]. These differences may be attributed to sample composition [9]; for example, studies with a predominance of gender-specific cancer types such as ovarian, cervical, uterine, and prostate cancers might explain these contradictory results in part due to the distribution of the study sample. A low level of education has also been associated with more fear of recurrence [15,16], although some studies indicate just the opposite [4,17].

Associations with other factors such as cancer type, stage, or treatment have also been investigated, although the results have not been conclusive [4,10]. Simard et al. [4] conducted a study comparing fear of cancer recurrence among breast, prostate, colorectal, and lung cancer survivors. The findings indicated that prostate cancer patients reported lower levels of fear of cancer recurrence compared to individuals with other cancer types. In her study of 2615 survivors of different types of cancer, van der Wal [18] found no differences between cancer types, but stage II subjects scored higher for fear of recurrence. This fear is affected by psychosocial factors, in addition to medical factors, which vary among individuals. Uncertainty, depression, and anxiety have been linked to high fear of recurrence in previous research [4,19,20]. Unlike other illnesses, the growth and development of cancer cells are often insidious and difficult to detect, leading to an intense sense of uncertainty, worry, and anxiety about the disease [21,22]. Social support also plays a significant role in fear of recurrence after cancer diagnosis and treatment [23,24]. Some studies have found that social support can alleviate this fear [24,25].

The literature on the relationship between fear of recurrence and sociodemographic, clinical, and psychosocial variables in advanced cancer patients is still inconclusive. To contribute to a more comprehensive understanding of the mechanisms related to fear of recurrence, the aim of this study is to analyze the sociodemographic, clinical, and psychosocial factors that may influence increased fear of recurrence in metastatic cancer patients before starting oncological treatment. Our hypothesis is that women, younger individuals, those with lung cancer, and those experiencing higher levels of depression, anxiety, uncertainty, and social support exhibit greater fear of recurrence.

## 2. Materials and Methods

### 2.1. Study Design and Population

**Figure 1:**
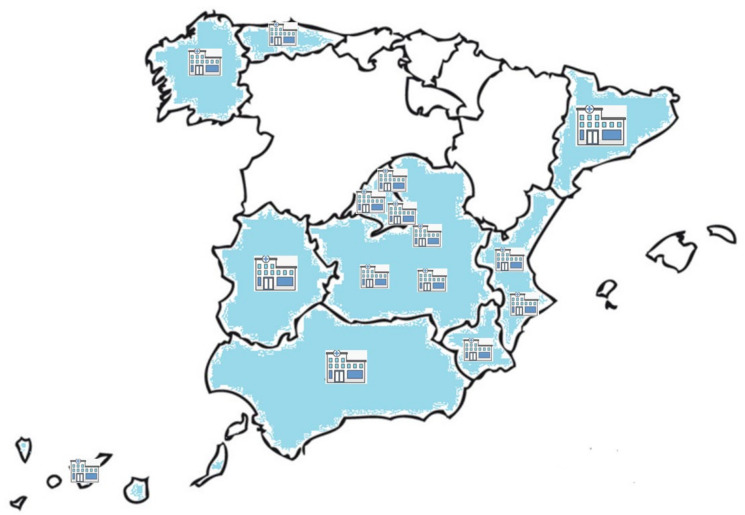
Distribution of hospital across the regions of Spain.

A prospective study, incorporating a cross-sectional analysis at a specific time point, was conducted in 15 hospitals across Spain. The research took place from February 2020 to September 2023, engaging multiple medical oncology services within these hospitals (refer to Figure 1 and Appendix A, Table A1). The objective was to collect data from advanced cancer patients who were not eligible for curative treatment. Patient selection took place during their initial visit to the medical oncologist, where they received information about their diagnosis, disease stage, and the systemic antineoplastic treatments available. To be eligible, participants had to be at least 18 years old, have a histopathological diagnosis of advanced cancer, and not be candidates for curative interventions. Several exclusion criteria were applied, including physical status, age, or comorbidity that could interfere with antineoplastic treatment, prior treatment for a different advanced cancer in the last two years, and any pre-existing condition that could hinder participation. Patients with cognitive impairment or severe general deterioration were likewise excluded. Ethical approval was obtained from the Ethical Review Committee of each participating institution, and this study was approved by the Spanish Agency of Medicines and Medical Devices (AEMPS) with identification code ES14042015. All participants provided informed consent, and data collection involved questionnaires, interviews, and the extraction of clinical information from medical records. The process was standardized across all participating centers, and patient data were collected from their respective institutions. Participation was voluntary and anonymous. Participants completed the written questionnaires at home and returned them at their next scheduled appointment. Medical oncologists used a web platform (www.neoetic.es) to update and collect data.

### 2.2. Description of Variables

Data collection methods involved the utilization of a standardized self-report form to collect sociodemographic information. Interviews were carried out by the attending oncologists, who also reviewed the medical records to obtain data concerning cancer diagnoses and treatments. During discussions about the benefits of systemic cancer treatment, oncologists provided subjects with questionnaires and encouraged them to complete these forms at home before commencing treatment. Patients subsequently returned these questionnaires to study personnel during their follow-up appointments. The questionnaires explicitly stated that participation was voluntary and anonymous.

The Cancer Worry Scale (CWS) is a widely used tool to assess fear of cancer recurrence [26]. The 6-item CWS inquiries about participants’ concerns regarding cancer recurrence and the impact of these concerns and is scored on a 4-point Likert scale (1 = ‘never’ to 4 = ‘almost always’). The total score ranges from 6 to 24, with higher scores indicating greater fear of recurrence. The scale was validated in Spanish populations, with a Cronbach’s alpha of 0.82 [27].

The Brief Symptom Inventory (BSI) [28] consists of 18 items divided into three dimensions (somatization, depression, and anxiety). Responses are measured on a five-point Likert-type scale, ranging from 0 (not at all) to 4 (very much). Raw scores are transformed into standardized T scores with a mean of 50 and standard deviation (SD) of 10 [28]. Internal consistency reliability in the cancer sample was strong (Cronbach’s alpha = 0.86, 0.86, and 0.91 for somatization, depression, and anxiety, respectively) [29].

A questionnaire employing the five-item Michel Uncertainty of Illness Scale (MUIS) [30] was used to appraise reactions to uncertainty, ambiguity, and the future. Items are scored on a 5-point Likert scale, yielding total scores of 5–25; higher scores correspond to greater uncertainty. Validation for Spanish populations yielded a Cronbach’s alpha of 0.91 for the scale [31].

Duke–UNC-11 Functional Social Support Questionnaire [32] was used to measure social support. This instrument assesses two dimensions of social support: confidential support (from people with whom the patient can communicate intimate feelings) and affective support (from those who express positive empathy toward the person). Each item is scored on a 5-point Likert scale, from 1 (much less than I would like) to 5 (as much as I would like). Scores range from 11 to 55; the higher the score, the more social support. In Spanish populations, the scale’s validation resulted in a Cronbach’s alpha coefficient of 0.94 [33].

### 2.3. Statistical Methods

Descriptive statistics and frequency distributions were calculated for demographic and clinical characteristics using SPSS version 26 (IBM SPSS Statistics for Windows, Armonk, NY, USA: IBM Corp.). To identify participants with similar fear of cancer recurrence patterns, a cluster analysis was conducted. Clustering variables comprised the fear of recurrence items. Given that this technique requires valid values for all variables, subjects with any missing CWS scores were eliminated. We carried out a k-means method using Euclidean distances between observations to estimate clusters and Ward’s hierarchical clustering method [34], in which the distance between two clusters is defined as the squared error criterion. In all instances, the distances were computed from the raw data to incorporate the elevation, scatter, and shape of the patients’ profiles [35]. A two-cluster solution was found to distinguish between low and high fear of recurrence. Analyses of variance (ANOVA) as well as chi-square analyses were performed to examine differences in demographic, clinical, and psychological characteristics among the fear of recurrence profiles. Eta squared (η^2^) was applied to calculate the effect size for continuous variables. Eta-squared ranges between 0 and 1, with η^2^~0.01 indicating a small, η^2^~0.06 a medium, and η^2^ > 0.14 a large effect size [35]. Multivariable logistic regression models were determined to identify and assess predictors of fear of recurrence; covariates included age, sex, educational level, and estimated survival. Odds ratios (ORs) and *p*-values were calculated and presented. A *p*-value of <0.05 was deemed statistically significant.

## 3. Results

### 3.1. Sociodemographic and Clinical Characteristics

Table 1 displays the sociodemographic and clinical characteristics of the 1195 study participants included in the analysis. The average age was 65.8 ± 11 years, with 52% being men. Most were married and had an intermediate level of education. The most common primary tumor sites were digestive (42%), bronchopulmonary (31%), and breast (12%), and the majority were diagnosed with metastatic disease (80% versus 20%). Chemotherapy was the most frequent treatment (51%), followed by immunotherapy (7%) and targeted therapies (6%). At the time of diagnosis, more than half of the participants had an Eastern Cooperative Oncology Group (ECOG) status of >2 (55%) and an estimated 11-month survival in most cases (55% versus 45%).

### 3.2. Fear of Recurrence Profiles and Clinical–Demographic Characteristics

We utilized a k-means method using Euclidean distances between observations to estimate clusters and Ward’s hierarchical clustering method. A two-cluster solution was found to distinguish between low and high degrees of fear of recurrence. All patients reported experiencing some level of fear, which was self-reported and categorized as low in 28% of cases (*n* = 332) and high in 72% (*n* = 863), based on their degrees of fear of recurrence.

Analyzing the relationship between fear of recurrence profiles and clinical and demographic characteristics of the study population, we found that women (*X*^2^ = 9.492, *p* = 0.002), individuals <55 years of age (*X*^2^ = 10.341, *p* = 0.016), those with a primary level of education (*X*^2^ = 10.812, *p* = 0.001), and those with a worse prognosis of less than 12-month survival (*X*^2^ = 4.497, *p* = 0.034) exhibited greater fear of recurrence than men, people aged ≥65 years, and those with a better prognosis. No statistically significant correlation was detected with marital status, employment, tumor site, histology, stage, performance status, or treatment modality (Table 1).

### 3.3. Characteristics Related to Patients’ Fear of Recurrence Profiles

When looking for relationships between fear of recurrence profiles and the psychosocial symptoms assessed using the scales (BSI, MUIS, DUFSSQ), we found that cancer patients with greater degrees of fear of recurrence scored higher for depression (*M* = 64.5 vs. *M* = 58.8; η^2^ = 0.138), anxiety (*M* = 66.2 vs. *M* = 59.2; η^2^ = 0.152), somatic symptoms (*M* = 65.6 vs. *M* = 62.7; η^2^ = 0.028), and uncertainty (*M* = 14.7 vs. *M* = 12.8; η^2^ = 0.048), and they expressed that they had greater social support (*M* = 42.8 vs. *M* = 41.3; η^2^ = 0.008) (Table 2).

Multivariate analyses revealed that younger patients (OR = 1.52; 95% CI [1.12–2.07]), those with a survival of <12 months (OR = 1.45; 95% CI [1.07–1.96]), higher scores on depression (OR = 1.10; 95% CI [1.06–1.13]), anxiety (OR = 1.09; 95% CI [1.06–1.13]), greater uncertainty (OR = 1.09; 95% CI [1.05–1.13]), and higher social support (OR = 1.05; 95% CI [1.03–1.07]) were significantly more likely to have fear of recurrence than older patients, and patients with greater survival expectancy, lower levels of anxiety, depression, uncertainty, and lower social support (Table 3).

## 4. Discussion

Fear of cancer recurrence is a widely shared concern among cancer patients. The aim of this study was to assess the sociodemographic, clinical, and psychological factors contributing to increased fear of recurrence in individuals with metastatic cancer prior to the start of oncological treatment. We hypothesized that women, younger patients, those with lung cancer, and those experiencing higher degrees of depression, anxiety, uncertainty, and social support would exhibit greater fear of recurrence. The results obtained partially supported our hypotheses.

In this study, approximately 28% of the sample reported high levels of fear of recurrence, similar to a previous review that found one in five patients experiencing a high degree of fear of recurrence [36]. Fear of recurrence was greater in women, younger patients, and those with a primary education, also consistent with previous research [8,9,15,16]. Fear of recurrence is greater in women due to their caregiving role and may be related to concerns about not being able to fulfill their responsibilities or becoming a burden to their loved ones [8,9]. Regarding age, the younger the cancer patient, the higher the reported fear of recurrence [4,37]. In younger subjects, fear of recurrence may be related with disruptions to their plans, and individuals with a primary education may lack access to medical information [4,9,15,16]. Furthermore, several studies have demonstrated that both men and women have cognitive biases in risk assessment, influencing their fear of recurrence [38]. Women tend to perceive risks as more threatening than men [8,39], and young individuals and those with a primary education may have a more catastrophic perception of risk given their lack of experience and knowledge [4,39].

In our study, the fear of recurrence was similar across all cancer types. Findings related to cancer type and fear of recurrence are contradictory. Some studies suggest that lung, breast, and melanoma cancer patients have higher fear of recurrence scores, while prostate cancer patients score lower [10,36,39]. Other studies indicate that the type of cancer has a limited and less-than-expected effect on fear of recurrence [9]. Some cancers are sex-specific, such as ovarian, cervical, uterine, and prostate cancer [40], which could lead to sex-biased results [9,40]. The same issue has been raised regarding age [37]. Research with a sample consisting of a higher percentage of young adult patients showed that they have significantly higher levels of fear of recurrence compared to those that included a broader age range [40].

The subgroup of participants with a shorter life expectancy had higher fear of recurrence scores. This may be because these individuals are more aware of the severity of their condition and the limitations in terms of treatment options and prospects for a cure. This cognizance can increase their fear of recurrence, as they understand that their disease is more difficult to control and treat and that the chances of a complete cure are low. Few studies have analyzed the relationship between life expectancy and fear of recurrence, but most of the ones that do exist suggest that patients who believe they have a longer life expectancy may experience more fear of recurrence [41,42].

Our study revealed that the relationships between fear of recurrence and selected psychosocial factors were as expected, based on the existing literature [4,37]. We found that patients who exhibited more pronounced symptoms of anxiety, somatization, and depression, as well as those with greater social support, reported higher fear of recurrence, which was confirmed in the multivariate analysis. These findings are consistent with earlier research that has noted the association between depressive symptoms and fear of recurrence [4,19,43]. Moreover, uncertainty and anxiety have also been seen to be associated with this fear of recurrence [4,20]. This study also found that being younger, having a less favorable prognosis, and having a lower educational level correlated with greater fear of recurrence, similar to the conclusions of other studies [3,39,44]. Nevertheless, no significant association was observed between sex and fear of recurrence in the multivariate model. This may be because our study sample consisted primarily of individuals with lung and colon cancer, two types of cancer that are relatively common in both sexes.

Few studies have explored the relationship between social support and fear of recurrence in advanced cancer. Social support encompasses emotional, practical, and caregiving provided by family, friends, and healthcare professionals with the aim of improving patients’ quality of life and well-being [25]. In our study, patients with extensive support networks exhibited a heightened fear of recurrence, likely influenced by the responsibility towards family members, especially children or dependents. This suggests that social support does not uniformly alleviate fear, warranting further investigation into how dependent family members influence fear of recurrence.

This study has several limitations related to its design, cohort composition, and methods. Data were derived from cross-sectional surveys, assessing fear of recurrence at a single time point, which impedes the ability to draw conclusions about trajectories and causal relationships. Moreover, it relied on self-reported data, which raises concerns about potential self-report bias. The inclusion of additional observational methods, such as medical reports or ratings from peers or family members, could provide added value and mitigate the potential for self-reporting bias. It is important to note that this study population consisted of Spanish patients with advanced cancer, limiting the generalizability of the findings to other disease stages or demographic groups.

Healthcare professionals can address mild distress by explaining the risks of recurrence and normalizing the fear surrounding it. Our study suggests that indicators such as psychological distress, uncertainty, and social support, along with a younger age, worse prognosis, and primary education, can help identify individuals more likely to experience a great deal of fear of recurrence. By combining screening tools and conversations with doctors, this knowledge can enable patients to be connected with interventions proven to reduce fear, such as group and individual cognitive–behavioral therapy, gratitude interventions, and specialized nurse counseling [45,46]. However, further research is needed to improve detection and referral and to identify evidence-based interventions for patients with advanced cancer.

## 5. Conclusions

In this study of subjects with advanced cancer, approximately one-third of the participants reported a high level of fear of recurrence. Our data, based on point-in-time surveys, suggest that psychological distress, uncertainty, and social support correlate with increased fear of recurrence. The surprising association between social support and fear of recurrence warrants further investigation. Future studies conducted with samples of resected cancer patients and/or using different time points to explore the potential causal relationship between social support and fear of recurrence are needed. These findings contribute to our understanding of the predictors of fear of recurrence and underscore the importance of the well-being of patients with advanced cancer.

## Figures and Tables

**Table 1:** Differences in demographic and clinical characteristics among the fear of recurrence profiles (*n* = 1195).

Variable	Total Sample *n* (%) 1195 (100%)	Low Fear of Recurr. *n* (%) 332 (28%)	High Fear of Recurr. *n* (%) 863 (72%)	*X* ^2^	*p*-Value
Sex					
Male	667 (56)	209 (63)	458 (53)	9.492	0.002
Female	528 (44)	123 (37)	405 (47)		
Age					
<55	173 (14)	32 (10)	141 (16)	10.341	0.016
55–64.9	322 (27)	86 (26)	236 (27)		
65–75	455 (38)	138 (41)	315 (37)		
>75	247 (21)	76 (23)	171 (20)		
Marital status					
Married or partnered	810 (68)	225 (68)	585 (68)	0.001	0.996
Not partnered	385 (32)	107 (32)	278 (32)		
Educational level					
Primary	563 (47)	131 (40)	100 (50)	10.812	<0.001
High school or more	632 (53)	201 (60)	431 (50)		
Employment					
Unemployed	759 (64)	225 (68)	534 (62)	3.508	0.061
Employed	435 (36)	107 (32)	328 (38)		
Tumor site					
Bronco-pulmonary	366 (31)	97 (29)	269 (31)	1.069	0.785
GI	497 (42)	145 (44)	352 (41)		
Breast	137 (12)	39 (12)	98 (11)		
Others	195 (16)	51 (15)	144 (17)		
Histology					
Adenocarcinoma	772 (65)	215 (65)	557 (65)	.005	0.944
Others	423 (35)	117 (35)	306 (35)		
Stage					
Locally advanced	238 (20)	68 (21)	170 (20)	0.092	0.761
Dis. metastases (IV)	957 (80)	264 (79)	693 (80)		
Type of treatment					
Chemotherapy	612 (51)	168 (51)	444 (51)	2.820	0.588
Immunotherapy	82 (7)	26 (8)	56 (7)		
Targeted therapies	66 (6)	20 (6)	46 (5)		
Others	430 (36)	118 (36)	317 (37)		
ECOG					
0 or 1	462 (39)	138 (42)	324 (38)	1.636	0.201
2 or more	733 (61)	194 (58)	539 (63)		
Estimated survival					
<12 months	535 (45)	132 (40)	403 (47)	4.497	0.034
≥12 months	659 (55)	199 (60)	460 (53)		

**Table 2:** Differences in baseline psychosocial characteristics and fear of recurrence profiles.

	Low Fear of Recurr. *n* (%) 332 (28%)		High Fear of Recurr. *n* (%) 863 (72%)				
	Mean	SD	Mean	SD	F	*p*	Eta-Squared
Psychological distress (BSI)							
Depression	58.8	5.4	64.5	6.6	190.44	0.001	0.138
Anxiety	59.2	6.5	66.2	7.8	213.83	0.001	0.152
Somatization	62.7	7.2	65.6	7.8	34.177	0.001	0.028
Illness uncertainty (MUIS)	12.8	4.2	14.7	3.9	58.780	0.001	0.047
Social support (DUFSSQ)	41.3	8.9	42.8	6.9	9.092	0.001	0.008

Abbreviation: BSI: Brief Symptom Inventory; MUIS, Michel Uncertainty in Illness Scale; DUFSSQ, Duke-UNC Functional Social Support Questionnaire; SD, standard deviation.

**Table 3:** Multivariate logistic regression of factors variables associated with high fear of recurrence.

Variable	Odds Ratio	95% CI		
		Low	High	*p*-Value
Sex: male	0.824	0.605	0.220	1.123
Educ: primary	0.984	0.971	0.029	0.998
Survival: <12 months	1.451	1.072	0.016	1.964
Age	1.528	1.128	0.006	2.070
Depression	1.100	1.062	0.000	1.139
Anxiety	1.096	1.064	0.000	1.128
Somatization	0.963	0.941	0.002	0.987
Illness uncertainty	1.093	1.052	0.000	1.134
Social support	1.052	1.031	0.000	1.074
Constant	0.000		0.000	

## Data Availability

Data are contained within the article.

## Appendix A

**Table A1:** List of 15 centers participating in this study.

(1) Hospital Universitario de Canarias, Tenerife
(2) Hospital Universitario La Paz, Madrid
(3) Hospital Universitario Central de Asturias, Oviedo
(4) Complejo Hospitalario Universitario de Ourense, Orense
(5) Hospital Universitario Infanta Leonor, Madrid
(6) Consorcio Hospital General Universitario de Valencia, Valencia
(7) Hospital General Virgen de la Luz, Cuenca
(8) Hospital Provincial de Castellón, Castellón
(9) Hospital General Universitario de Elche, Elche
(10) Hospital Universitario Clínico San Carlos, Madrid
(11) Hospital San Pedro de Alcántara, Cáceres
(12) Hospital Universitario Morales Meseguer, Murcia
(13) Hospital Quironsalud Sagrado Corazón de Sevilla, Sevilla
(14) Hospital General Universitario Santa Lucia, Cartagena
(15) Hospital General Universitario de Ciudad Real, Ciudad Real

## References

[1] Bergerot C.D., Philip E.J., Bergerot P.G., Siddiq N., Tinianov S., Lustberg M. (2022). Fear of Cancer Recurrence or Progression: What Is It and What Can We Do about It?. Am. Soc. Clin. Oncol. Educ. Book Am. Soc. Clin. Oncol. Annu. Meet..

[2] Lebel S., Ozakinci G., Humphris G., Thewes B., Prins J., Dinkel A., Butow P. (2017). Current state and future prospects of research on fear of cancer recurrence. Psychooncology.

[3] Smith A.B., Sharpe L., Thewes B., Turner J., Gilchrist J., Fardell J.E., Girgis A., Tesson S., Descallar J., Bell M. (2018). Medical, demographic and psychological correlates of fear of cancer recurrence (FCR) morbidity in breast, colorectal and melanoma cancer survivors with probable clinically significant FCR seeking psychological treatment through the ConquerFear study. Support. Care Cancer.

[4] Simard S., Thewes B., Humphris G., Dixon M., Hayden C., Mireskandari S., Ozakinci G. (2013). Fear of cancer recurrence in adult cancer survivors: A systematic review of quantitative studies. J. Cancer Surviv..

[5] Champagne A., Ivers H., Savard J. (2018). Utilization of health care services in cancer patients with elevated fear of cancer recurrence. Psychooncology.

[6] Williams J.T.W., Pearce A., Smith A.B. (2021). A systematic review of fear of cancer recurrence related healthcare use and intervention cost-effectiveness. Psychooncology.

[7] Ozdemir S., Ng S., Wong W.H.M., Teo I., Malhotra C., Mathews J.J., Joad A.S., Hapurachchi T., Toung P.N., Bhatnagar S. (2022). Advanced Cancer Patients Prognostic Awareness and Its Association with Anxiety, Depression and Spiritual Well-Being: A Multi-Country Study in Asia. Clin. Oncol..

[8] Aminisani N., Nikbakht H.-A., Shojaie L., Jafari E., Shamshirgaran M. (2022). Gender Differences in Psychological Distress in Patients with Colorectal Cancer and Its Correlates in the Northeast of Iran. J. Gastrointest. Cancer.

[9] Pang C., Humphris G. (2021). The Relationship between Fears of Cancer Recurrence and Patient Gender: A Systematic Review and Meta-Analysis. Front. Psychol..

[10] Luigjes-Huizer Y.L., Tauber N.M., Humphris G., Kasparian N.A., Lam W.W.T., Lebel S., Simard S., Smith A.B., Zachariae R., Afiyanti Y. (2022). What is the prevalence of fear of cancer recurrence in cancer survivors and patients? A systematic review and individual participant data meta-analysis. Psychooncology.

[11] Séguin Leclair C., Lebel S., Westmaas J.L. (2019). The relationship between fear of cancer recurrence and health behaviors: A nationwide longitudinal study of cancer survivors. Health Psychol..

[12] Gemmill R., Sun V., Ferrell B., Krouse R.S., Grant M. (2010). Going with the Flow Quality-of-Life Outcomes of Cancer Survivors with Urinary Diversion. J. Wound Ostomy Cont. Nurs..

[13] Luo X., Li W., Yang Y., Humphris G., Zeng L., Zhang Z., Garg S., Zhang B., Sun H. (2020). High Fear of Cancer Recurrence in Chinese Newly Diagnosed Cancer Patients. Front. Psychol..

[14] Jeon M.Y.M.L., Sung T., Han M., Shin Y., Kim W., Chung K., Shong J., Kim W. (2019). Quality of life in patients with papillary thyroid microcarcinoma managed by active surveillance or lobectomy: A cross-sectional study. Thyroid.

[15] Liu Y., Pérez M., Schootman M., Aft R.L., Gillanders W.E., Jeffe D.B. (2011). Correlates of fear of cancer recurrence in women with ductal carcinoma in situ and early invasive breast cancer. Breast Cancer Res. Treat..

[16] Meissner V.H., Olze L., Schiele S., Ankerst D.P., Jahnen M., Gschwend J.E., Herkommer K., Dinkel A. (2021). Fear of cancer recurrence and disease progression in long-term prostate cancer survivors after radical prostatectomy: A longitudinal study. Cancer.

[17] Hamrick N., Diefenbach M.A. (2006). Religion and spirituality among patients with localized prostate cancer. Palliat. Support. Care.

[18] Van de Wal M., van de Poll-Franse L., Prins J., Gielissen M. (2016). Does fear of cancer recurrence differ between cancer types? A study from the population-based PROFILES registry. Psychooncology.

[19] Walburg V., Rueter M., Lamy S., Compaci G., Lapeyre-Mestre M., Laurent G., Despas F. (2019). Fear of cancer recurrence in Non- and Hodgkin lymphoma survivors during their first three years of survivorship among French patients. Psychol. Health Med..

[20] Mell C.A., Jewett P.I., Teoh D., Vogel R.I., Everson-Rose S.A. (2022). Psychosocial predictors of fear of cancer recurrence in a cohort of gynecologic cancer survivors. Psychooncology.

[21] Mishel M.H. (1997). Uncertainty in Acute Illness. Annu. Rev. Nurs. Res..

[22] Cruz-Castellanos P., Gil-Raga M., Jiménez-Fonseca P., Ghanem I., Hernández R., Piera-Molons N., Cano J.M., Gallego-Martinez A., García-Torralba E., Calderon C. (2022). Uncertainty and hope in relation to anxiety and depression in advanced lung cancer. BMJ Support. Palliat. Care.

[23] Thompson T., Pérez M., Kreuter M., Margenthaler J., Colditz G., Jeffe D.B. (2017). Perceived social support in African American breast cancer patients: Predictors and effects. Soc. Sci. Med..

[24] Yu Z., Sun D., Sun J. (2022). Social Support and Fear of Cancer Recurrence Among Chinese Breast Cancer Survivors: The Mediation Role of Illness Uncertainty. Front. Psychol..

[25] Zheng W., Hu M., Liu Y. (2022). Social support can alleviate the fear of cancer recurrence in postoperative patients with lung carcinoma. Am. J. Transl. Res..

[26] Custers J.A.E., Kwakkenbos L., van de Wal M., Prins J.B., Thewes B. (2018). Re-validation and screening capacity of the 6-item version of the Cancer Worry Scale. Psycho-oncology.

[27] Cabrera E., Zabalegui A., Blanco I. (2011). Versión española de la Cancer Worry Scale (Escala de Preocupación por el Cáncer: Adaptación cultural y análisis de la validez y la fiabilidad). Med. Clin..

[28] Derogatis L. (2001). BSI 18, Brief Symptom Inventory 18: Administration, Scoring and Procedures Manual.

[29] Calderón C., Ferrando P.J., Lorenzo Seva U., Hernández R., Oporto-Alonso M., Jiménez Fonseca P. (2020). Factor structure and measurement invariance of the Brief Symptom Inventory (BSI-18) in cancer patients. Int. J. Clin. Health Psychol..

[30] Mishel M.H. (1983). Adjusting the Fit: Development of Uncertainty Scales for Specific Clinical Populations. West. J. Nurs. Res..

[31] Brito-Brito P.R., García-Tesouro E., Fernández-Gutiérrez D.Á., García-Hernández A.M., Fernández-Gutiérrez R., Burillo-Putze G. (2018). Validación de la Escala de Incertidumbre ante la Enfermedad en pacientes y acompañantes que acuden a un servicio de urgencias. Emergencias.

[32] Broadhead W.E., Gehlbach S.H., De Gruy F.V., Kaplan B.H. (1988). The Duke UNC Functional Social Support Questionnaire. Med. Care.

[33] Ayala A., Rodríguez-Blázquez C., Frades-Payo B., Forjaz M.J., Martínez-Martín P., Fernández-Mayoralas G., Rojo-Pérez F. (2012). Propiedades psicométricas del Cuestionario de Apoyo Social Funcional y de la Escala de Soledad en adultos mayores no institucionalizados en España. Gac. Sanit..

[34] Joe H., Ward J. (1963). Hierarchical Grouping to Optimize an Objective Function. J. Am. Stat. Assoc..

[35] Pierce C.A., Block R.A., Aguinis H. (2004). Cautionary note on reporting eta-squared values from multifactor ANOVA designs. Educ. Psychol. Meas..

[36] Smith A.B., Costa D., Galica J., Lebel S., Tauber N., van Helmondt S.J., Zachariae R. (2020). Spotlight on the Fear of Cancer Recurrence Inventory (FCRI). Psychol. Res. Behav. Manag..

[37] Lim E., Humphris G. (2020). The relationship between fears of cancer recurrence and patient age: A systematic review and meta-analysis. Cancer Rep..

[38] Butow P., Kelly S., Thewes B., Hruby G., Sharpe L., Beith J. (2015). Attentional bias and metacognitions in cancer survivors with high fear of cancer recurrence. Psychooncology.

[39] Galica J., Maheu C., Brennestuhl S., Townsley C., Metcalfe K. (2021). Examining Predictors of Fear of Cancer Recurrence Using Leventhl’s Commonsens Model. Cancer Nurs..

[40] Stephens C., Westmaas J.L., Kim J., Cannady R., Stein K. (2016). Gender differences in associations between cancer-related problems and relationship dissolution among cancer survivors. J. Cancer Surviv..

[41] Loh K.P., Mohile S., Epstin R., McHugh C., Flannery M., Culakova E., Lei L., Wells M., Gilmore N., Babu D. (2019). Willingness to Bear Adversity and Beliefs about the Curability of Advanced Cancer in Older Adults. Cancer.

[42] Mackenzie L.J., Carey M.L., Suzuki E., Sanson-Fisher R.W., Asada H., Ogura M., D’Este C., Yoshimura M., Toi M. (2018). Agreement between patients’ and radiation oncologists’ cancer diagnosis and prognosis perceptions: A cross sectional study in Japan. PLoS ONE.

[43] Liu J., Peh C.-X., Simard S., Griva K., Mahendran R. (2018). Beyond the fear that lingers: The interaction between fear of cancer recurrence and rumination in relation to depression and anxiety symptoms. J. Psychosom. Res..

[44] Ng D.W.L., Kwong A., Suen D., Chan M., Or A., Ng S.S., Foo C.C. (2019). Fear of cancer recurrence among Chinese cancer survivors: Prevalence and associations with metacognition and neuroticism. Psychooncology.

[45] Hall D.L., Luberto C.M., Philpotts L.L., Song R., Park E.R., Yeh G.Y. (2018). Mind-body interventions for fear of cancer recurrence: A systematic review and meta-analysis. Psychooncology.

[46] Tauber N.M., O’Toole M.S., Dinkel A., Galica J., Humphris G., Lebel S., Maheu C., Ozakinci G., Prins J., Shapre L. (2019). Effect of Psychological Intervention on Fear of Cancer Recurrence: A Systematic Review and Meta-Analysis. J. Clin. Oncol..

